# Daily land use regression estimated woodsmoke and traffic pollution concentrations and the triggering of ST-elevation myocardial infarction: a case-crossover study

**DOI:** 10.1007/s11869-017-0537-1

**Published:** 2017-12-11

**Authors:** David Q. Rich, Mark J. Utell, Daniel P. Croft, Sally W. Thurston, Kelly Thevenet-Morrison, Kristin A. Evans, Frederick S. Ling, Yilin Tian, Philip K. Hopke

**Affiliations:** 1 Department of Public Health Sciences, University of Rochester School of Medicine and Dentistry, 265 Crittenden Boulevard CU 420644, Rochester, NY 14642 USA; 2 Department of Environmental Medicine, University of Rochester School of Medicine and Dentistry, 601 Elmwood Avenue Box EHSC, Rochester, NY 14642 USA; 3 Division of Pulmonary and Critical Care Medicine, Department of Medicine, University of Rochester Medical Center, 601 Elmwood Avenue Box 692, Rochester, NY 14642 USA; 4 Department of Biostatistics and Computational Biology, University of Rochester School of Medicine and Dentistry, 265 Crittenden Boulevard Box 630, Rochester, NY 14642 USA; 5 Division of Cardiology, Department of Medicine, University of Rochester Medical Center, 601 Elmwood Avenue, Paul N. Yu Heart Center, Rochester, NY 14642 USA; 6 Center for Air Resources Engineering and Science, Clarkson University, Box 5708, Potsdam, NY 13699 USA

**Keywords:** Air pollution, Myocardial infarction, Delta-C, Black carbon, Land use regression

## Abstract

Prior work has reported acute associations between ST-elevation myocardial infarction (STEMI) and short-term increases in airborne particulate matter. Subsequently, the association between STEMI and hourly measures of Delta-C (marker of woodsmoke) and black carbon (marker of traffic pollution) measured at a central site in Rochester, NY, were examined, but no association was found. Therefore, land use regression estimates of Delta-C and black carbon concentrations at each patient’s residence were developed for 246 STEMI patients treated at the University of Rochester Medical Center during the winters of 2008–2012. Using case-crossover methods, the rate of STEMI associated with increased Delta-C and BC concentration on the same and previous 3 days was estimated after adjusting for 3-day mean temperature and relative humidity. Non-statistically significant increased rates of STEMI associated with interquartile range increases in concentrations of BC in the previous 2 days (1.10 μg/m^3^; OR = 1.12; 95% CI 0.93, 1.35) and Delta-C in the previous 3 days (0.43 μg/m^3^; OR = 1.16; 95% CI 0.96, 1.40) were found. Significantly increased rates of STEMI associated with interquartile range increases in concentrations of BC (1.23 μg/m^3^; OR = 1.04; 95% CI = 0.87, 1.24) or Delta-C (0.40 μg/m^3^; OR = 0.94; 95% CI = 0.85, 1.09) on the same day were not observed likely due, in part, to temporal misalignment. Therefore, sophisticated spatial-temporal models will be needed to minimize exposure error and bias by better predicting concentrations at individual locations for individual hours, especially for outcomes with short-term responses to air pollution (< 24 h).

## Introduction

Short-term increases in ambient particulate matter (PM) concentrations over hours and days have been associated with various manifestations of cardiovascular disease, including myocardial infarction (MI) (Mustafic et al. 2012; Gardner et al. 2014). Previously, Gardner et al. (2014) observed a significant 18% increase in the rate of ST-elevation myocardial infarction (STEMI), but not non-ST-elevation myocardial infarction (NSTEMI), associated with each 7.1 μg/m^3^ increase in fine particulate pollution (< 2.5 μm diameter; PM_2.5_) concentrations in the previous hour among patients in Monroe County, NY. Others have subsequently reported triggering of STEMI, but not NSTEMI, by short-term increases in PM_2.5_ concentrations (Pope et al. 2015; Zhang et al. 2016). However, it is unknown which PM_2.5_ constituent(s)/source(s) trigger STEMI.

Previously, Wang et al. (2012a, b) estimated that up to 30% of wintertime PM_2.5_ is contributed by woodsmoke in Rochester, NY. Therefore, Evans et al. (2017) examined whether increased hourly concentrations of source-specific PM_2.5_, including Delta-C (marker of woodsmoke) (Wang et al. 2011a) and black carbon (marker of traffic pollution) (Suglia et al. 2008), measured at a single, central, monitoring station in Rochester in the previous 1, 12, 24, 48, 72, and 96 h were associated with an increased rate of STEMI. However, neither pollutant was associated with an increased rate of STEMI in the previous 96 h (Evans et al. 2017). In that analysis, all study subjects’ pollutant concentrations were taken from the same monitoring station no matter how far they lived from it, resulting in exposure error and likely effect underestimation (Evans et al. 2017). This exposure error may be fairly substantial for Delta-C and black carbon, which have greater spatial variability in concentration than PM_2.5_ (Larson et al. 2007; Su et al. 2007; Wang et al. 2011b). Wang et al. (2011b) measured BC at a number of locations in Rochester and concluded that one central monitoring site may not adequately represent the actual residential wood combustion particle exposure over the whole urban area. Thus, this exposure error could induce substantial attenuation of the estimated associations between STEMI and these pollutants. Use of central site measured concentrations alone may not be sufficient to observe air pollution-mediated health effects if they do exist.

Thus, a land use regression (LUR) model was developed to predict daily concentrations of black carbon and Delta-C at the residence of each STEMI patient during the study period, to provide specific estimates of subjects’ actual exposures to these pollutants (Su et al. 2015). Using these daily LUR estimates of black carbon and Delta-C concentrations, and the same data set of STEMI events, it was hypothesized that increased concentrations of black carbon and Delta-C on the same day as the STEMI would be associated with an increased rate of STEMI.

## Methods

### Study population

The study population was described previously by Evans et al. (2017). Briefly, patients treated at the Cardiac Catheterization Laboratory (Cath Lab) at the University of Rochester Medical Center in Rochester, NY, for STEMI between November 1 and April 30 from 2008 to 2012, who were Monroe County, NY residents, and for whom symptom onset time was recorded were included in the analysis (*n* = 246). Using American College of Cardiology/American Heart Association guidelines at the time of Cath Lab admittance, STEMI was diagnosed as ST segment elevation on the electrocardiogram of > 1 mm in ≥ 2 contiguous precordial leads, or ≥ 2 adjacent limb leads, or new or presumed-new left bundle branch block in the presence of angina or anginal equivalent. If a patient was admitted for multiple MIs during the study, each STEMI was included in the analysis if it was at least 3 days after the previous MI. Information on patient demographic and clinical characteristics, including smoking, history of MI, and other comorbidities (peripheral artery disease, heart failure, diabetes, dyslipidemia, and hypertension) were also retained. All study activities were approved by the University of Rochester Research Subjects Review Board.

### Air pollution and meteorology measurements

Black carbon, Delta-C, and particulate matter < 2.5 μm (PM_2.5_) concentrations, temperature, and relative humidity measured between November 1, 2008 and April 30, 2012 at a New York State Department of Environmental Conservation (DEC) site in Rochester, NY, were used to drive the model predictions. Using a two-wavelength (370 and 880 nm) aethalometer (Model AE22, Magee Scientific, Inc., Berkeley, CA), black carbon was estimated using the 880-nm measurements, with Delta-C calculated as the difference between the 370-nm and 880-nm measurements. Delta-C is a surrogate for woodsmoke because it was highly correlated with levoglucosan (Wang et al. 2011a), the compound commonly used as a marker of wood combustion particles (Simoneit 2002). Hourly PM_2.5_ concentrations were measured continuously using a tapered element oscillating microbalance (TEOM; model 1400ab, ThermoFisher, Franklin, MA). Ambient temperature and relative humidity were measured in 5-min intervals at the DEC site and provided as hourly averages. The residential addresses of each study subject were geocoded (ArcGis Version-10.3.1.©Esri, Redlands, CA), and daily black carbon and Delta-C concentrations were estimated using the land use regression model developed for the cold period (November 1 to April 30) in Monroe County, NY (Su et al. 2015).

### Study design and statistical analyses

The time-stratified case-crossover design (Levy et al. 2001) used previously in studies of ambient air pollution and MI (Mustafic et al. 2012; Gardner et al. 2014; Pope et al. 2015; Evans et al. 2017) was used in this study. Mean concentrations of Delta-C, BC, temperature, and relative humidity were calculated for the same day (lag day 0) and previous 2 days (lag days 0–1), 3 days (lag days 0–2), and 4 days (lag days 0–3). Conditional logistic regression models were used to estimate the rate (and 95% confidence intervals) of STEMI associated with interquartile range (IQR) increases in pollutant concentrations during each lag period, adjusting for the 3-day mean temperature and relative humidity using natural splines (2 degrees of freedom). SAS version 9.3 (SAS Institute, Inc., Cary, NC) was used to construct all data sets and perform descriptive analyses, and R version 3.0.1 (R Foundation for Statistical Computing, Vienna, Austria) was used for all conditional logistic regression models.

## Results

Most patients (52%) were between 50 and 69 years old (mean age ± standard deviation = 61.8 ± 15.5 years). Subjects were primarily male (68%) and non-Hispanic white (80%). Approximately one third were smokers (37%) and 76% were either overweight or obese (BMI ≥ 25 kg/m^2^; Table 1). The median and mean ± standard deviation of daily black carbon concentrations on control days during the study period were 0.69 μg/m^3^ and 1.10 ± 1.25 μg/m^3^. For Delta-C, they were 0.10 μg/m^3^ and 0.34 ± 0.64 μg/m^3^. For case periods, the median and mean ± standard deviation of daily black carbon concentrations were 0.68 μg/m^3^ and 1.10 ± 1.29 μg/m^3^, while for Delta-C, they were 0.11 μg/m^3^ and 0.31 ± 0.57 μg/m^3^. Daily LUR-estimated black carbon and Delta-C were minimally correlated (Spearman *r* = 0.32). For comparison, the mean ± standard deviation ambient PM_2.5_ concentrations at the Rochester monitoring station were 7.8 ± 4.1 μg/m^3^ for case periods and 7.8 ± 4.0 μg/m^3^ for control periods.

**Table 1:** Subject characteristics (*N* = 246 STEMI patients)

	*N*	%
Age		
< 50 years	45	18
50–59 years	63	26
60–69 years	67	27
70–79 years	45	18
≥ 80 years	26	11
Male (*n* = 245)^1^	166	68
Race/ethnicity (*n* = 235)^1^		
Non-Hispanic white	187	80
Non-Hispanic black	33	14
Hispanic	6	3
Other	9	4
Clinical presentation		
Prior cardiovascular disease (*n* = 171)^1,2^	4	2
Prior myocardial infarction (*n* = 244)^1^	42	17
Prior percutaneous intervention (*n* = 245)^1^	42	17
Prior coronary artery bypass (*n* = 245)^1^	7	3
Prior peripheral artery disease (*n* = 244)^1^	11	5
Prior heart failure (*n* = 243)^1^	8	3
Smoker (*n* = 235)^1^	87	37
Hypertension (*n* = 242)^1^	178	74
Dyslipidemia (*n* = 243)^1^	149	61
Diabetes (*n* = 231)^1^	49	21
BMI (*n* = 281)^1^		
Normal weight (BMI < 25)	58	26
Overweight (BMI 25.0–29.9)	83	38
Obesity: class I (BMI 30.0–34.9)	51	23
Obesity: class II (BMI 35.0–39.9)	22	10
Obesity: class III (BMI ≥40)	6	3
Mean (SD)	28.8 (5.2)	

Percentages do not always add up to 100% due to rounding

^1^Number of events/patients with data available for a given characteristic

^2^As indicated by “Prior CVD” in Cath Lab record, or a prior MI, prior PCI, or prior CABG

Although imprecise and not statistically significant, increased rates of STEMI were associated with interquartile range increases in concentrations of black carbon in the previous 2 days (1.10 μg/m^3^; OR = 1.12; 95% CI 0.93, 1.35) and Delta-C in the previous 3 days (0.43 μg/m^3^; OR = 1.16; 95% CI 0.96, 1.40). No increased rates of STEMI associated with Delta-C or black carbon concentrations on the same day, as hypothesized, were observed (Table 2). In a sensitivity analysis using the land use regression estimated Delta-C and black carbon concentrations averaged over the same days, and averaged over a 500-m radius around the location of each residence (Su et al. 2015), similar size effects were found (Table 2). In a second sensitivity analysis, we re-ran the same models, including the ambient PM_2.5_ concentration for the same lag time to determine if any effect of BC or Delta-C was independent of total fine particle mass. However, after adjustment for PM_2.5_, there was little change in the rate of STEMI associated with each IQR increase in Delta-C in the previous 3 days (OR = 1.16, 95% CI = 0.96, 1.40) or black carbon in the previous 2 days (OR = 1.12; 95% CI = 0.93, 1.35).

**Table 2:** Rate of STEMI associated with interquartile range increases in mean pollutant concentrations, estimated using land use regression, by lagged averaging time

	Lag days averaged	Interquartile range	*N*	Odds ratio	95% CI
Delta-C (μg/m^3^) At each residence	0	0.40	245	0.96	(0.85, 1.09)
	0, 1	0.39	243	1.10	(0.95, 1.28)
	0, 1, 2	0.43	240	1.16	(0.96, 1.40)
	0, 1, 2, 3	0.42	240	1.09	(0.87, 1.36)
Black carbon (μg/m^3^) At each residence	0	1.23	245	1.04	(0.87, 1.24)
	0, 1	1.10	244	1.12	(0.93, 1.35)
	0, 1, 2	1.09	241	1.05	(0.84, 1.31)
	0, 1, 2, 3	1.04	240	1.03	(0.81, 1.31)
Delta-C (μg/m^3^) Average in 500 m around each residence	0	0.40	245	0.98	(0.85, 1.13)
	0, 1	0.39	244	1.11	(0.93, 1.33)
	0, 1, 2	0.43	241	1.12	(0.91, 1.38)
	0, 1, 2, 3	0.42	240	1.05	(0.83, 1.33)
Black carbon (μg/m^3^) Average in 500 m around each residence	0	1.23	245	1.07	(0.89, 1.28)
	0, 1	1.10	244	1.08	(0.89, 1.31)
	0, 1, 2	1.09	241	0.98	(0.79, 1.23)
	0, 1, 2, 3	1.04	240	0.96	(0.75, 1.23)

All models adjusted for 3-day average temperature and relative humidity

## Discussion

In a case-crossover study examining the triggering of ST-elevation myocardial infarction by short-term increases in daily ambient black carbon (marker of traffic pollution) and Delta-C (marker of woodsmoke) concentrations estimated at each study subject’s residence using a land use regression model, increased but imprecise and not statistically significant rates of STEMI were found to be associated with increased concentrations of these source-specific PM constituents in the previous 2 and 3 days. However, an increased rate of STEMI associated with increased concentrations of these PM constituents was not found on the same day, as originally hypothesized.

Previously, in a similar case-crossover study, Gardner et al. (2014) reported an increased rate of STEMI associated with increased PM_2.5_ (measured at a fixed site in Rochester, NY) concentrations in the previous 1 h, suggesting that mechanistically, the hour before symptom onset for STEMI is an important time to estimate PM concentrations. A second analysis by Evans et al. (2017) examined whether increases in concentrations of source-specific PM constituents (black carbon and Delta-C) also triggered STEMI in the previous few hours. However, increased rates of STEMI associated with increased pollutant concentrations in the previous 1, 12, 24, 48, 72, or 96 h were found. In that analysis, the associations with pollutant concentrations were examined for the same lag times (previous few hours before symptom onset) as the Gardner et al. (2014) analysis, providing reasonable temporal alignment with the estimated symptom onset time. However, there was likely to be spatial misalignment (Peng and Bell 2010) since all subjects were assigned pollutant values from that fixed monitoring station, no matter how far they lived from that monitoring station and Wang et al. (2011b) had shown spatial inhomogeneity in the BC and Delta-C concentrations. This spatial misalignment and exposure error likely resulted in bias towards the null and underestimates of effect, perhaps obscuring any increased rates of STEMI associated with central site measured black carbon and Delta-C concentrations if they truly existed (Evans et al. 2017).

Weichenthal et al. (2017) reported a significant 6% increase in the odds of MI hospital admissions associated with increased concentrations of PM_2.5_ in the previous 3 days among elderly subjects (≥ 65 years of age), but not young subjects (< 65), among residents of three regions of British Columbia, Canada. Further, the strongest associations were observed in the cold season when monthly mean biomass burning contributions to PM_2.5_ (based on ambient levoglucosan/PM_2.5_) were in the highest tertile (OR = 1.19; 95% CI = 1.04, 1.36). Our Delta-C findings, although not statistically significant, are consistent with this study.

There has been limited work examining cardiovascular health effects of woodsmoke exposure (Naeher et al. 2007; Sigsgaard et al. 2015). Studies done in occupationally exposed firefighters and using controlled woodsmoke exposures have had mixed results, with some, but not all, reporting increased pulmonary and systemic inflammation and hemostatic responses (Barregard et al. 2006, 2008; Forchhammer et al. 2012; Hejl et al. 2013; Swiston et al. 2008; Tan et al. 2000). However, some studies have reported null findings for acute cardiovascular events in populations exposed to forest fire smoke (Delfino et al. 2009; Hanigan et al. 2008; Henderson et al. 2011; Johnston et al. 2007; Sigsgaard et al. 2015). In cities where woodsmoke PM is a large component of total winter PM, increased concentrations in the winter have been associated with increased cardiorespiratory admissions (Fairley 1999; Sanhueza et al. 2009; Schwartz et al. 1993). Other community-wide and in-home intervention studies have reported improved levels of inflammatory, endothelial function, blood pressure, and myocardial ischemia biomarkers, as well as reduced cardiorespiratory mortality associated with reduced woodsmoke exposures and concentrations (Allen et al. 2009; Johnston et al. 2013; McCracken et al. 2007, 2011). Previously, Croft et al. (2017) reported increased fibrinogen levels associated with increased Delta-C concentrations in the previous 12 h. Those findings are consistent with the studies showing cardiovascular responses to woodsmoke.

In this analysis, a land use regression model (Su et al. 2015) was used to estimate 24-h average Delta-C and black carbon concentrations at each subject’s residence providing greater spatial alignment than the previous analyses (Gardner et al. 2014; Evans et al. 2017). However, concentrations could only be estimated for the day of symptom onset, and not the specific hour(s), resulting in temporal misalignment. This temporal exposure error likely resulted in a bias towards the null and underestimates of effect. Further, the rate of STEMI associated with increased land use regression estimated black carbon and Delta-C concentrations in the previous few hours could not be estimated. Thus, to properly assess the rate of STEMI (and likely other acute cardiopulmonary events with a short triggering time) associated with short-term (< 24 h) increases in concentrations of source-specific PM constituents (e.g., black carbon and Delta-C), future studies should have a larger sample size (to increase statistical power) and use more sophisticated spatial-temporal models that can minimize both temporal and spatial misalignment. These models should be able to accurately estimate hourly pollutant concentrations at each subject’s residence. As investigators use epidemiology studies (e.g., panel, cohort, and case-control studies) to examine biologic mechanisms of air pollution-mediated acute cardiorespiratory events that may act on hourly time scales rather than days, weeks, and months, these more sophisticated spatial-temporal models will be needed.

### Source of funding

This work was supported by grants from the New York State Energy Research and Development Authority (contract no. 32971) and National Institute of Environmental Health Sciences (grant no. P30 ES001247). Daniel Croft and Kristin Evans were supported by a National Institutes of Health training grant (T32-HL066988-1).
